# Development and evaluation of cardiovascular disease risk prediction models for patients with type 2 diabetes

**DOI:** 10.1038/s41598-026-45129-5

**Published:** 2026-03-31

**Authors:** Yang Yang, Tian Liu, Che-Yi Liao, Sun Ju Lee, Esmaeil Keyvanshokooh, Hui Shao, Mary Beth Weber, Francisco J. Pasquel, Gian-Gabriel P. Garcia

**Affiliations:** 1 H. Milton Stewart School of Industrial and Systems Engineering, Georgia Institute of Technology, Atlanta, GA USA; 2 Freeman College of Management, Bucknell University, Lewisburg, PA USA; 3 Department of Information and Operations Management, Mays Business School, Texas A&M University, College Station, TX USA; 4 Hubert Department of Global Health, Rollins School of Public Health, Emory University, Atlanta, GA USA; 5 Division of Endocrinology, Metabolism, and Lipids, Department of Medicine, Emory University School of Medicine, Atlanta, GA USA; 6 Department of Industrial and Systems Engineering, University of Washington, Seattle, WA USA; 7 Department of Computer Science and Engineering, Texas A&M University, College Station, TX USA

**Keywords:** Risk equation development, Survival modeling, Cardiovascular diseases, Type 2 diabetes, Fairness evaluation, Cardiology, Diseases, Endocrinology, Health care, Medical research, Risk factors

## Abstract

To facilitate treatment decisions in people at risk of Cardiovascular Disease (CVD), several risk equations such as the Pooled Cohort Equations and Predicting Risk of Cardiovascular Disease Events (PREVENT) equations have been developed to estimate CVD risk for primary prevention patients. However, it is unclear whether these equations achieve high predictive accuracy and fairness in patients with type 2 diabetes (T2D), and whether a T2D-specific risk equation is needed. Accordingly, we developed a Weibull Accelerated Failure Time (AFT) survival model for predicting the 3-year CVD risk in 23,795 patients with T2D from the All of Us dataset, using sociodemographic information, physical measurements, medication, and CVD history. Among patients without CVD history, our Weibull AFT (vs. PREVENT) achieved a greater C-index (0.646 vs. 0.465), greater Concordance Fractions (0.610–0.674 vs. 0.541–0.600), and comparable Concordance Imparity (0.006 vs. 0.002) across sex and race/ethnicity (0.065 vs. 0.058) subgroups. Our findings highlight the need for a T2D-specific CVD risk equation and demonstrate the value of diverse datasets for developing fair and accurate predictive models.

**Supplementary Information:**

The online version contains supplementary material available at 10.1038/s41598-026-45129-5.

## Introduction

Cardiovascular disease (CVD) is a leading cause of death worldwide. In the United States, national CVD mortality rose during the COVID-19 pandemic and has remained high; more than 200,000 CVD deaths occurred from 2020 to 2022, representing a 9% increase over projections based on historical CVD deaths from 2010 to 2019^1^. Notably, adults with type 2 diabetes (T2D) experience CVD at elevated rates due to commonly co-occurring risk factors such as hypertension and dyslipidemia. Moreover, CVD is the leading cause of morbidity and mortality in this population^2^. To facilitate timely prevention and treatment among at-risk patients, accurate estimation of CVD risk is critical.

To facilitate treatment decisions for at-risk patients, significant efforts have been made to develop CVD risk prediction models. In 2013, the American Heart Association (AHA) and the American College of Cardiology (ACC) developed the pooled cohort equations (PCEs), which are sex and race-specific models to estimate the 10-year atherosclerotic CVD (ASCVD) event risk for African-American and White men and women aged 40 to 79 years without previous CVD history^3,4^. The PCEs are based on age, total cholesterol, high-density lipoprotein cholesterol, systolic BP, diabetes mellitus, and current smoking status. Although the PCEs were endorsed by the 2019 AHA/ACC Primary Prevent Guidelines, experts raised many concerns. First, the PCEs did not account for heart failure, which is a rising prevalent CVD subtype^5,6^. In addition, the PCEs do not generalize to other race and ethnicity groups such as Asians and Hispanics, which were not included in its derivation^7^. Moreover, the PCEs were developed over a decade ago and thus may not reflect recent population-level changes in risk factors and better preventive treatments^8,9^.

With the goal of more accurate and equitable CVD risk assessment, in 2023, the AHA Cardiovascular-Kidney-Metabolic Scientific Advisory Group developed and externally validated the Predicting Risk of Cardiovascular Disease EVENTs (PREVENT) risk equations using 25 datasets that contain a total of 6 million participants collected between 1992 and 2017^10,11^. PREVENT focused on predicting the risk of ASCVD, heart failure, and total CVD for primary prevention patients, providing sex-specific 10-year risk estimates for individuals 30–79 years of age and 30-year risk estimates for individuals 30–59 years of age. Enhanced models that consider Hemoglobin A1c, urine albumin-to-creatinine ratio, and social deprivation index were also developed.

While risk equations such as the PCEs and PREVENT have positively impacted clinical care for CVD, there remain several opportunities to further advance the utility of these tools. First, there is a dearth of CVD risk equations specifically for adults with T2D. Accordingly, there is limited evidence on whether existing risk equations such as PREVENT can adequately predict risk of CVD among adults with T2D compared to specific risk equations developed for this population. Second, it is unclear whether widely used risk equations generate fair predictions across different demographic subgroups. For example, despite the extensive external validation performed on PREVENT, there exists limited literature which examines its predictive fairness across different racial subgroups^12^. Lastly, existing risk equations focused on primary prevention and thus may not generalize to patients with a history of CVD.

To bridge these gaps, we developed a Weibull Accelerated Failure Time (AFT) survival model using a diverse cohort of patients with T2D from the National Institutes of Health (NIH) All of Us dataset to predict 3-year risk of CVD among adults with T2D. We assess this model across several dimensions, including variable importance, model calibration, predictive accuracy, and fairness. Then, we benchmark against PREVENT across these metrics. Our results demonstrate that our Weibull AFT model outperforms PREVENT in calibration, predictive accuracy, and group fairness when validated against a diverse cohort of adult patients with T2D.

## Methods

### Data source

We leveraged the All of Us dataset from the NIH^13^, a longitudinal cohort study aiming to advance precision medicine. The All of Us dataset integrated diverse individual-level data from electronic health records (EHRs), participant-derived information from surveys, physical measurements, biospecimens, and wearables. As of August 2024, the program has enrolled over 832,000 participants, covering a diverse population from historically underrepresented groups in biomedical research (> 80%), e.g., racial and ethnic minorities (45%)^14^. Every participants in the All of Us program completes a survey at enrollment which gathers information about a participant’s demographics, lifestyle, personal and family health history, healthcare access and utilization, and social determinants of health among other topics.

### Data preprocessing

Using the cloud-based Research Workbench, we followed four steps to preprocess the data. First, we selected patients with T2D and extracted demographic characteristics (age, sex, race, ethnicity), socio-economic factors (e.g., employment status, insurance coverage), clinical features (CVD/kidney disease history), medication history, and relevant biomarkers (see Table 1 for more information). These predictors were chosen for their clinical relevance and availability in primary care. See eAppendix A for additional details on data preprocessing. Second, we filtered out patients younger than 40 to align with AHA guidelines, which recommend risk estimation for adults aged 40 to 74, considering that CVD typically occurs in older populations^15^. Third, we used Random Forest to impute missing continuous data (< 6%) and one-hot encoded the categorical variables. We split the data into 80% training and 20% testing sets and normalized continuous variables before model fitting. Fourth, we added interaction terms to capture the non-additive effects between pre-survey medication history and biomarkers (see details in the supplementary risk calculation spreadsheet).

Next, we formatted our data so that it was amenable to survival analysis, i.e., time-to-event modeling. Since patients may develop competing diseases (e.g., kidney disease) before CVD, our study focuses on time-to-first-event (i.e., *duration*) modeling^16^. That is, for patients who develop CVD, the duration is the interval between the survey and diagnosis dates. For right-censored patients^17^, the duration is the time between the survey and last clinical visit unless they are censored due to a competing event. If a competing disease occurs before CVD, we use the interval between the survey date and the competing disease diagnosis date.

### Survival model development

Similarly to PREVENT, our primary outcome was risk of CVD event, comprised of myocardial infarction (i.e., heart attack), stroke, and heart failure. Since 96% of durations in our data were 3 years or fewer, we focused on 3-year CVD risk. Moreover, we opted to use a Weibull Accelerated Failure Time (Weibull AFT) model for CVD risk prediction because of its intuitive interpretation in survival analysis, as demonstrated in previous works^18,19^. In our analysis, we fit a Weibull AFT model to the training data with demographic characteristics, socio-economic factors, clinical features, medication history, and relevant biomarkers as predictors. See eAppendix B for additional details on comparisons between our modeling variables and those in PREVENT. Also, see eAppendix C for an analysis on the impact of race and ethnicity as predictors and eAppendix D for an analysis of our model trained only on the subset of patients with history of CVD. Finally, since PREVENT was developed for patients without CVD history, we performed all comparisons between PREVENT and the Weibull AFT model on a subset of patients without CVD history in the testing set as well as the entire testing set. Comparisons were made on calibration, predictive accuracy, and fairness as are described in further detail below. 10-year risks from PREVENT were approximately converted to 3-year risks (see eAppendix E).

### Variable importance

We evaluated the impact of predictors on the performance of our Weibull AFT model using permutation importance^20^. Permutation importance measures each predictor’s impact on predictive accuracy by perturbing its values while keeping others constant and calculating the resulting change in performance as measured by area under the receiver operating characteristic curve (AUC). To ensure reliable estimates, we bootstrapped 100 iterations, sampling the training data, training a model, calculating baseline AUC, and determining importance scores for each iteration. We report the average permutation importance scores, where a higher importance indicates stronger model dependence on that variable to make accurate predictions.

### Model calibration

We evaluated the calibration of our Weibull AFT model and PREVENT by comparing predicted 3-year CVD risks with observed event rates across sex subgroups and race and ethnicity subgroups in the test set using risk calibration plots^21^. For each subgroup, we calculated both the Weibull AFT model’s predicted 3-year CVD risk and the scaled risk from PREVENT equations. After grouping patients into deciles based on predicted risk scores, we computed the mean predicted risk and observed event rate for each subgroup. Plotting observed rates against mean predicted risks revealed any discrepancies in risk estimation across predicted risk levels.

### Accuracy evaluation

We evaluated the predictive accuracy of the Weibull AFT model and PREVENT using the concordance index (C-index) in the testing set. The C-index describes the proportion of time that, when two patients are randomly selected, the patient with a shorter time-to-CVD event is given a higher risk score than the patient with the longer time-to-CVD event. Greater C-index values are indicative of better predictive accuracy.

### Fairness evaluation

We compared the fairness of the Weibull AFT model with PREVENT across different sex subgroups and race and ethnicity subgroups using the Concordance Fraction (CF) and Concordance Imparity (CI)^22^. Compared to other existing fairness metrics that are commonly used in binary classification, CF and CI account for censorship in the data to ensure rigorous fairness evaluation in survival analysis^23^. Concretely, CF measures individual-level prediction accuracy within each subgroup by comparing model predictions with true outcomes. It excludes incomparable pairs where the shorter duration is censored or both durations are censored with identical survival times. CF for a subgroup is computed as the proportion of all correctly predicted pairs to all comparable pairs. A higher CF for a subgroup indicates better prediction accuracy for that group. We remark that CF and C-index have similar interpretations. However, a subgroup-specific C-index would only include comparisons within that subgroup and would fail to include comparisons to individuals outside of that subgroup.

Our second metric, CI, measures the disparity in prediction performance across subgroups. It is calculated as the absolute difference between CFs of the two subgroups that result in the largest difference. A lower CI score indicates a more fair model, suggesting that the model does not significantly favor one subgroup over another.

## Results

### Description of study population

Table 1 summarizes our training (*N* = 19,036) and testing datasets (*N* = 4759).

**Table 1:** Characteristics of study participants (n = 23,795) with type 2 Diabetes from All of Us dataset. Categorical features are represented as n (%) and continuous features are represented as mean (SD).

Characteristics	Training data (*n* = 19,036)	Testing data (*n* = 4759)
Demographics		
Age, years		
40–49	2097 (11.77%)	501 (11.25%)
50–59	4767 (26.76%)	1171(26.29%)
60–69	6098 (34.23%)	1556 (34.93%)
> 69	4854 (27.25%)	1227 (27.54%)
Sex		
Female	10,757 (56.51%)	2657 (55.83%)
Male	7836 (41.16%)	1980 (41.61%)
Other	443 (3.00%)	122 (2.56%)
Race and ethnicity		
White	9081 (47.70%)	2317 (48.69%)
Black	5167 (27.14%)	1256 (26.39%)
Hispanic	3548 (18.64%)	896 (18.83%)
Other	1240 (6.51%)	290 (6.09%)
Socio-economic factors		
Education level: College graduate or advanced degree	5692 (29.90%)	1456 (30.59%)
Employment Status, employed	5043 (26.49%)	1240 (26.06%)
Health Insurance, current	17,910 (94.08%)	4490 (94.34%)
Living Situation: Stable House Concern	3093 (16.25%)	799 (16.79%)
Income, annual income		
Less than 10k	3046 (16.00%)	791 (16.62%)
Between 10k and 100k	9808 (51.52%)	2414 (50.72%)
More than 100k	2064 (10.84%)	531 (11.16%)
Other	3758 (19.74%)	1023 (21.50%)
Housing type		
Apartment	86 (0.45%)	17 (0.36%)
Single Family	1791 (9.42%)	474 (9.96%)
Mixed	858 (4.51%)	215 (4.52%)
Townhouse	485 (2.55%)	130 (2.73%)
No information	15,816 (83.08%)	3943 (82.85%)
Psychological features		
Religious practice: frequent	927 (4.87%)	222 (4.67%)
Neighborhood trust: high	17,325 (91.01%)	4360 (91.62%)
Neighborhood drug use concern: high	15,107 (79.36%)	3769 (79.20%)
Speaks Non-English Language	576 (3.03%)	139 (0.03%)
Clinical features		
Tobacco usage, 100 in lifetime	8719 (45.80%)	2188 (45.08%)
History of cardiovascular disease	4699 (24.68%)	1108 (23.28%)
History of kidney disease	4198 (22.05%)	1044 (21.94%)
BMI, kg/m^2^	33.52 (6.09)	33.53 (6.90)
Blood pressure		
Systolic, mmHg	131.23 (14.95)	131.09 (14.46)
Diastolic, mmHg	76.10 (10.83)	76.11 (12.75)
Heart rate, bpm	78.43 (12.25)	78.27 (12.33)
Medication history within 1 year of survey date		
Aspirin	5677 (29.82%)	1407 (29.57%)
Cholesterol regulation medications	3944 (20.98%)	1046 (21.98%)
Diabetes medications		
Category 1 (non-insulin oral med + GLP1)	7639 (40.13%)	2027 (42.59%)
Category 2 (Category 1 + 1-shot BASAL)	1348 (7.08%)	298 (6.26%)
Category 3 (multiple daily injections)	2554 (13.42%)	608 (12.78%)
Category 4 (no diabetes medications)	7495 (39.37%)	1826 (38.37%)
Statin	11,626 (61.07%)	2893 (60.79%)
Anti-hypertension medications	14,376 (75.52%)	3579 (75.20%)
Laboratory measurements		
Calcium (mg/dL)	9.5 (130.65)	9.5 (132.41)
Cholesterol in HDL (mg/dL)	47.91 (11.12)	47.91 (11.12)
Creatine (mmol/L)	58.11 (126.11)	59.82 (127.59)
Magnesium (mg/dL)	1.94 (0.15)	1.94 (0.15)
Potassium (mmol/L)	63.06 (128.11)	63.88 (129.67)
Triglyceride (mg/dL)	156.38 (87.91)	158.50 (100.16)
Total Cholesterol (mg/dL)	167.60 (39.01)	168.12 (39.65)
Hemoglobin A1c (%)	7.16 (1.48)	7.13 (1.45)
Non-HDL cholesterol (mg/dL)	119.69 (37.61)	120.22 (38.16)
Glomerular filtration rate (mL/min/1.73 m^2^)	78.62 (9.61)	78.94 (9.00)
Duration of CVD event		
< 1 year	6090 (32.00%)	1477 (31.03%)
1 year–2 years	3064 (16.10%)	799 (16.37%)
2 years–3 years	5506 (28.92%)	1370 (28.79%)
3 years–4 years	3581 (18.81%)	943 (19.82%)
> 4 years	795 (4.18%)	190 (3.99%)
Outcomes		
Heart failure	2598 (13.65%)	604 (12.69%)
Stroke	1656 (8.70%)	413 (6.68%)
Heart attack	1113 (5.85%)	273 (5.74%)
Total CVD	5367 (28.19%)	1290 (27.11%)

### Model explanations

**Fig. 1:**
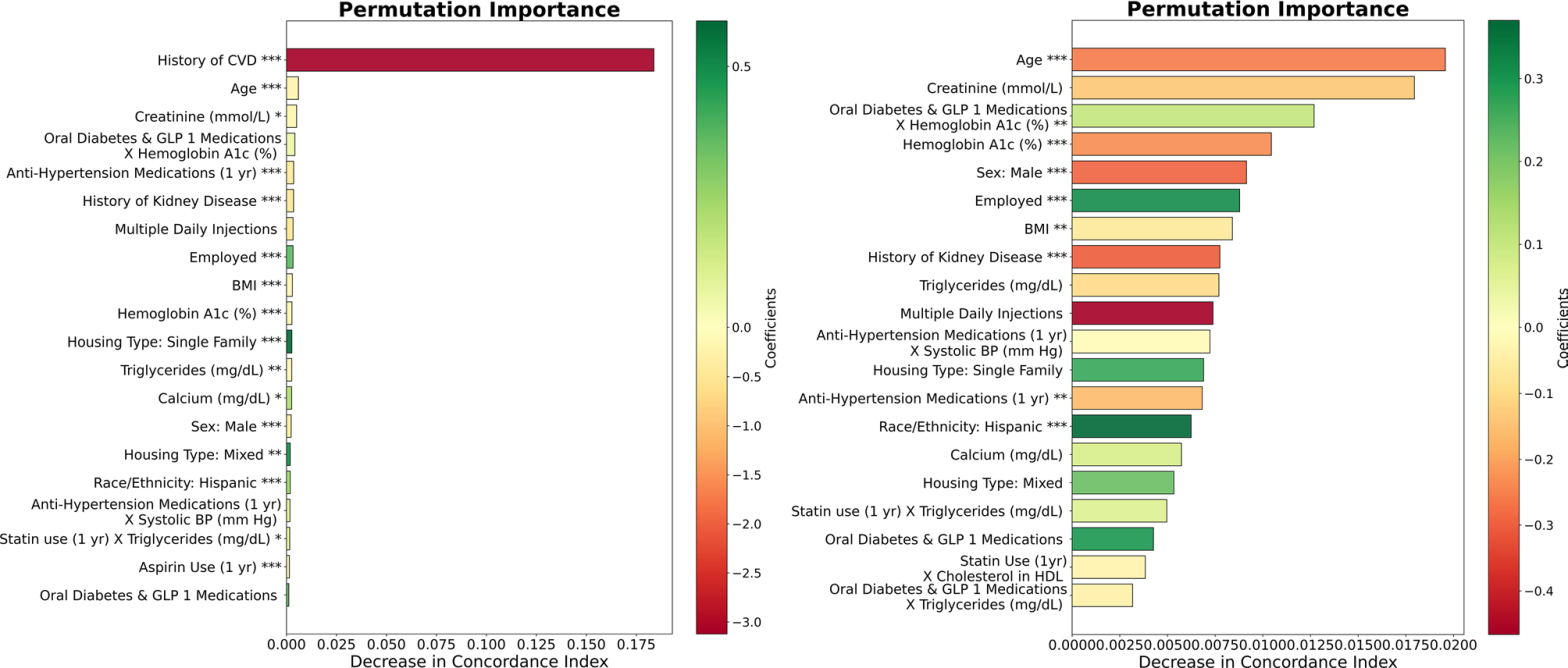
Permutation importance plots. Left: our Weibull AFT model when evaluated on all patients. Right: our Weibull AFT model when evaluated on patients without CVD history. Predictors are color-coded based on their variable coefficients to indicate whether they increase (red) or decrease (green) CVD risk. Note that the color scale is different in these two plots. Statistical significance of coefficients given by: *P < 0.05; **P < 0.01; ***P < 0.001.

Permutation variable importance plots are shown in Fig. 1. The color of the bars represents the positive or negative impact of each predictor on the predicted CVD risk. When evaluated on all patients, CVD history is the most important predictor in our developed model, followed by kidney disease-related predictors (e.g. Creatinine, Calcium, History of Kidney Disease). When evaluated on only patients without CVD history, age appears to be the most important factor. Importantly, for both model types, socio-demographic factors such as employment status, housing types, education level, and sex are among the top predictors.

### Model assessment–risk calibration

**Fig. 2:**
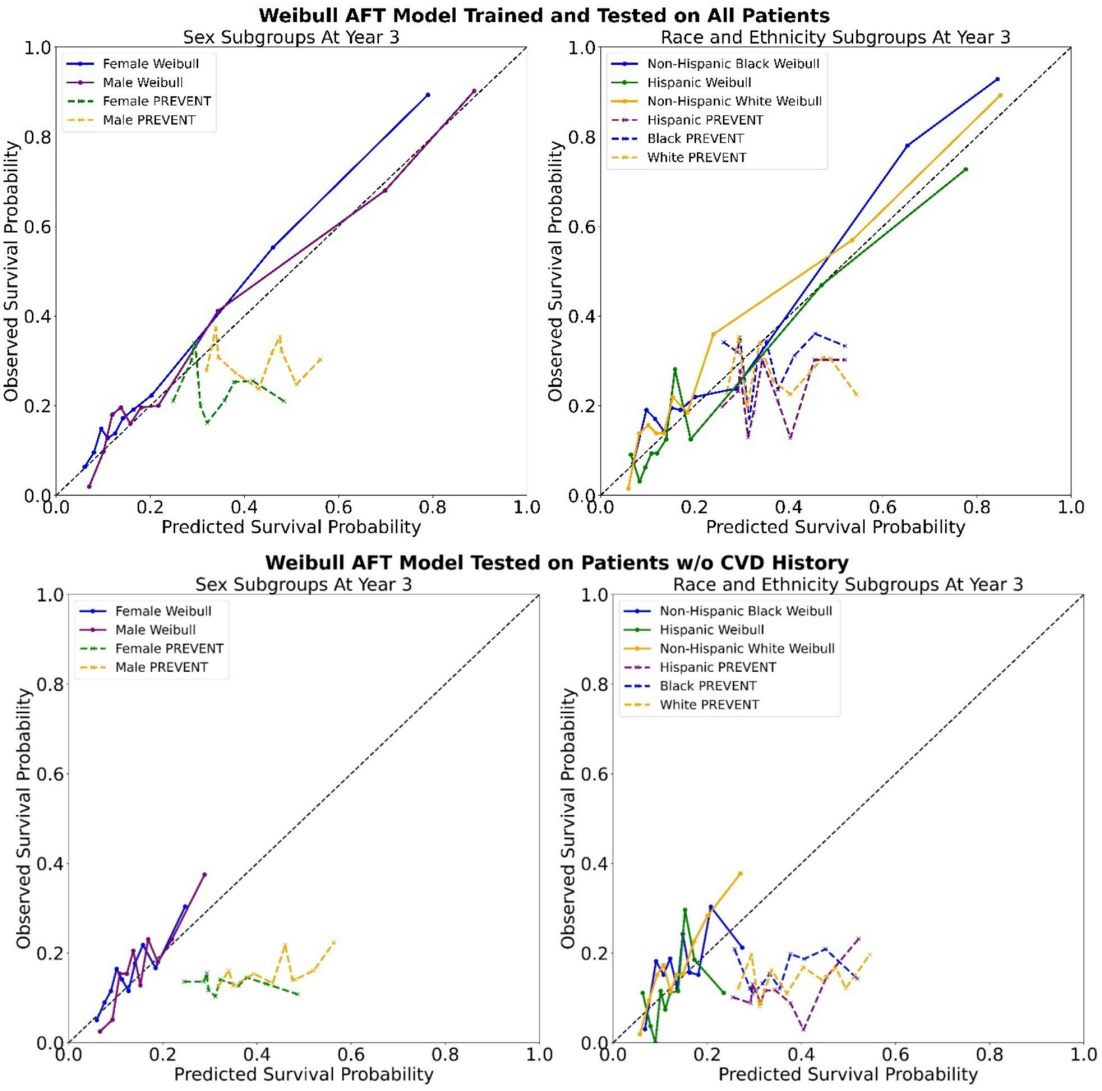
Three-year risk calibration plots for different sex subgroups and race and ethnicity subgroups comparing our developed Weibull AFT model with PREVENT when (1) testing on all patients (top) and (2) when testing on patients without CVD history (bottom). Ideal calibration is marked with a black dashed line.

Figure 2 (top) shows risk calibration plots for sex subgroups and race and ethnicity subgroups, indicating strong model calibration across the entire patient cohort. Additionally, our Weibull AFT model appears to have better calibration than PREVENT when testing on patients without CVD history (see Fig. 2, bottom). Notably, PREVENT over-estimates risk of CVD events in both analyses. We provide model coefficients and risk calculation examples for the Weibull AFT model in Supplement 2.

### Model assessment–predictive accuracy

When applied to the entire patient cohort in our testing set, our Weibull AFT model achieves a high C-index of 0.812, significantly outperforming PREVENT which achieves a C-index of 0.485. When comparing these models on the patients without CVD history in the testing set, the Weibull AFT model achieves a higher C-index of 0.646 compared to PREVENT’s 0.465.

### Model assessment–prediction fairness

Table 2 summarizes the CF and CI achieved by our Weibull AFT model and the PREVENT equations on different subgroups of data. When applied to all patients in the testing data, the Weibull AFT model achieves CF values ranging from 0.795 to 0.826 and a CI of 0.006 for the sex subgroups and 0.031 for the race and ethnicity subgroups. These values indicate better fairness than PREVENT, whose CF values range from 0.551 to 0.592 and CI equaling 0.019 and 0.041 for sex and race and ethnicity subgroups, respectively. Among patients without CVD history, Weibull AFT still achieves greater CF values than PREVENT (0.610–0.674 vs. 0.541–0.600), although the differences between the two models are smaller than when tested on the entire patient cohort. To this end, PREVENT achieved smaller CI values than the Weibull AFT for both sex (0.002 vs. 0.006) and race and ethnicity (0.058 vs. 0.065) subgroups, though these differences are small.

**Table 2:** Comparison of subgroup fairness metrics between our Weibull AFT model trained on all patients and PREVENT which was developed on patients without CVD history for primary prevention when evaluated on all patients and on patients without CVD history. We exclude other ethnicities (e.g., Asian) as they represent only 6% of our studied cohort, which may introduce large variability.

Metric	Testing set	Model	Sex		Race and ethnicity		
			Female	Male	Non-hispanic black	Hispanic	Non-hispanic white
Concordance fraction	All patients	Weibull	0.813	0.808	0.795	0.826	0.813
		PREVENT	0.574	0.554	0.551	0.592	0.559
	Patients w/o CVD history	Weibull	0.649	0.643	0.610	0.674	0.657
		PREVENT	0.568	0.569	0.541	0.600	0.572
Concordance imparity	All patients	Weibull	0.006		0.031		
		PREVENT	0.019		0.041		
	Patients w/o CVD history	Weibull	0.006		0.065		
		PREVENT	0.002		0.058		

## Discussion

Using NIH All of Us data, this study sought to investigate whether existing CVD risk prediction models can accurately and fairly estimate risk of CVD among patients with T2D. In particular, we developed a Weibull AFT model to estimate CVD risk among patients with T2D and compared its accuracy and fairness across sex and race and ethnicity subgroups to the PREVENT equations. Chiefly, our Weibull AFT model generally outperformed the established PREVENT equations on a large cohort of adults with T2D, both with and without history of CVD, in terms of calibration, predictive accuracy, and fairness on stratifications of this cohort by sex and race and ethnicity. This superior performance was upheld even when the Weibull AFT model was trained without the use of race and ethnicity as a predictor (see Appendix C) and when it was trained only on the subset of patients without history of CVD (see eAppendix D).

Our results support the need for risk equations designed specifically for adults with T2D. While many CVD risk factors overlap between our cohort of patients with T2D and the general population, we note that markers of kidney disease (e.g. history of kidney disease, creatinine, and calcium levels) emerged as some of the most important predictors in our model for patients with T2D. This finding is particularly significant because—while isolated kidney disease may not be a strong predictor of CVD in the general population—it does play a significant role for CVD risk prediction among patients with T2D, a disease characterized by neurohormonal activation and chronic subclinical inflammation^24–29^. Consistent with the current understanding of cardiorenal syndromes, kidney dysfunction in this population may contribute to increased risk of CVD through heightened cardiac remodeling, vascular injury, inflammatory toxicity, and neurohormonal activation, in addition to volume overload^25,30^. The strong association between kidney disease and CVD risk in patients with T2D underscores the importance of monitoring renal function as part of CVD risk assessment in this population.

In further support of the need for T2D-specific risk equations, we found that the Weibull AFT model is better-calibrated among our cohort of T2D compared to PREVENT; PREVENT systematically over-estimated event risk, especially among lower-risk patients without prior CVD, while the Weibull AFT curves nearly trace the ideal line for both sex and race/ethnicity groups. This strong calibration is crucial for clinical decision support since accurately estimated probabilities may help avoid unnecessary interventions in lower-risk patients and ensure high-risk individuals are identified reliably.

We additionally emphasize that the Weibull AFT outperformed PREVENT in terms of predictive fairness. While the exact reason for this performance gap is difficult to pinpoint, we hypothesize that this result may be driven partly by the fact that the Weibull AFT was trained on NIH All of Us data, which over-represents under-represented racial groups compared to the 25 datasets used to derive PREVENT. Further, All of Us enabled us to include fine-grained socio-economic features (e.g. housing type, income levels, employment status, education backgrounds) in our model. Incorporating these detailed socio-economic factors allows us to capture the social determinants of health more effectively. To this end, our results confirm that these features achieve high feature importance, likely because they better characterize patients’ accessibility to medical treatment and environments which support healthy lifestyle choices, supported by findings in the literature^31–33^. This suggests that socioeconomic factors play a critical role in CVD risk and should be considered in risk prediction models to improve their accuracy and fairness.

Critically, our Weibull models included a patient’s race and ethnicity as a predictor. Indeed, whether including race as a predictor promotes equity remains an open question^34–38^. One perspective which was adopted by PREVENT is to remove race as a predictor since race is a social construct rather than biological predictor^10^. However, this *fairness through unawareness* approach has been shown to be ineffective in some cases since race is often strongly correlated with other features such as income level, education level, and other socio-economic status^34,39,40^. As such, we opted to investigate whether there was any utility in including race as a predictor and found that race is among the top predictors in terms of ensuring predictive accuracy. To ascertain these findings, we also performed a supplementary analysis (see eAppendix C) to determine if a Weibull AFT model which did not include race and ethnicity could perform comparably to our Weibull AFT model that did include race and ethnicity. We found that excluding race and ethnicity resulted in lower C-indices overall and lower CF values in nearly all patient subgroups compared to our Weibull AFT model that included race and ethnicity. In other words, our model including race and ethnicity is both more accurate overall and fairer for most patient subgroups included in the analysis. While these results suggest that it may be beneficial to include race and ethnicity as a predictor in CVD risk prediction models broadly, we caution that these results are specific to our study data and modeling approach; additional investigation is warranted to ascertain how these findings extend to cohorts beyond our study.

Additionally, we found that when our model was evaluated on all patients, history of CVD events was one of the most important factors for ensuring accurate CVD risk prediction, far outweighing other variables. Our findings are supported by existing literature which suggest a prior CVD event increases the likelihood of future CVD events^41–45^. When our model was evaluated on patients without CVD history, age was the most important factor. This finding is consistent with previous literature suggesting that older individuals are at higher CVD risk^45,46^.

Finally, we argue that CVD risk estimation models for people with T2D—including our own Weibull AFT model—have the potential to provide valuable clinical decision support for personalized CVD prevention and risk management^47–50^. In particular, these models can quantitatively estimate an individual patient’s CVD risk, which can help to characterize the magnitude of risk, support shared decision-making in the clinic, and reinforce adherence to preventive therapies. For example, T2D is often accepted as a CVD equivalent^51^. Thus, for patients with T2D and no prior CVD history, CVD risk estimates can help guide statin intensity (e.g., moderate vs. high-intensity statin therapy), prompt consideration of treatment intensification in higher-risk individuals, and facilitate discussions around comprehensive risk-factor modifications (e.g., lifestyle interventions, smoking cessation, and adherence to cardioprotective and renoprotective therapies).

While the size and diversity of the NIH All of Us dataset is certainly a strength of our analysis, we acknowledge that the generalizability of our results beyond this cohort may be limited because our model was evaluated using the unseen test split from the same All of Us dataset. In the future, we plan to validate our developed model using external datasets. Additionally, we did not include Apolipoprotein B (ApoB) in our analysis, even though it has been found to be a better predictor of CVD risk than LDL and non-HDL cholesterol^52^. While approximately 1300 patients have an ApoB measurement in the entire All of Us dataset, our study sample specifically did not include any of these patients. Accordingly, we could not incorporate it in our analysis. Future studies should include ApoB if it is sufficiently available in the dataset. Lastly, our work focuses on time-to-first-event modeling where we censor the data when patients develop competing diseases (e.g. kidney diseases) before any CVD outcomes. We plan to further study how other diseases might affect subsequent CVD risk, such as increased doctor visits and changes in medications.

## Conclusion

In conclusion, by leveraging the All of Us dataset, this study developed a Weibull AFT survival model for predicting the CVD risks of patients with T2D. Our model demonstrates better accuracy and fairness than the existing PREVENT equations across different demographic strata and serves as a foundation toward more equitable risk estimation of CVD among patients with T2D, supporting improved clinical decision-making and potentially reducing disparities in CVD outcomes.

## Supplementary Information

Below is the link to the electronic supplementary material.

Supplementary Material 1

Supplementary Material 2

## Data Availability

The dataset supporting the conclusions of this article is available from the All of Us Research Program. The data are not openly available. Access to the data requires registration, training, and compliance with the All of Us Research Program data usage policies. The underlying code for this study, including those used to extract and process the training/testing datasets and perform the analysis, is not publicly available but may be made available to qualified researchers on reasonable request from the corresponding author.
