# Supplementary material for: Development and evaluation of cardiovascular disease risk prediction models for patients with type 2 diabetes
Source: Sci Rep. 2026 Mar 31;16:15574. doi: 10.1038/s41598-026-45129-5 (PMC13187328; doi:10.1038/s41598-026-45129-5)
Supplement: Supplementary file 2 — Supplementary Material 2 [file 41598_2026_45129_MOESM2_ESM.pdf]

Development and Evaluation of Cardiovascular Disease Risk Prediction Models for Patients with Type 2 Diabetes (Supplementary Material)

In this supplementary material, we provide more analyses and discussions to support our main paper, including the following sections:

- eAppendix A: Cohort definition and feature extraction
- eAppendix B: Comparison of the risk predictors of our Weibull AFT model with PREVENT
- eAppendix C: Analysis on race and ethnicity as predictors for our Weibull AFT model
- eAppendix D: Comparing accuracy and fairness of our Weibull AFT model trained on patients without CVD history with PREVENT
- eAppendix E: Converting PREVENT's 10-year risk to 3-year risk

eAppendix A: Cohort definition and feature extraction

We considered medication history up to one year before the survey date to reflect recent treatments. Individual socio-economic variables were prioritized over a composite index (e.g. Social Deprivation Index or SDI) for clearer insights into healthcare access and financial stability. We defined each patient's index date as the date they completed the survey during clinical visit. To identify T2D cases, we required at least one T2D diagnosis recorded on or before that index date. History of diseases were drawn from the electronic health record prior the index date as well. On the index date itself, we pulled static variables such as demographic characteristics and socioeconomic factors. Finally, we extracted longitudinal data after the index date, namely relevant biomarkers and medication usage, to support subsequent outcome analyses.

eAppendix B: Comparison of the risk predictors of our Weibull AFT model with PREVENT

The following eTable 4 compares the predictors used in our Weibull model with that of PREVENT. Our model includes more fine-grained socio-economic factors, additional psychological factors, CVD and Kidney disease history than PREVENT. We also include race as a predictor in our model.

eTable 4. Comparison of predictors in our Weibull AFT model and PREVENT. Features included in the enhanced version of PREVENT are marked with *.

Predictors	Weibull AFT (ours)	PREVENT
Demographics	Age, Sex, Race and Ethnicity	Age, Sex
Socio-economic Factors	Education Level, Employment Status, Health Insurance, Living Situation, Income, Housing Type	Social Deprivation Index*
Psychological Factors	Religious Practice, Neighborhood Trust, Neighborhood Drug Usage Concern, Speaks non-English Language	None
Clinical Features	Tobacco Usage, History of Cardiovascular Disease, History of Kidney Disease, BMI, Blood Pressure, Heart Rate	Systolic Blood Pressure, Diabetes, Tobacco Usage, BMI
Drug Usage within 1 year	Aspirin, Cholesterol Regulation Medication, Diabetes Medications, Statin, Anti-hypertension Medication	Anti-hypertension, medication use (Antihtn), Statin
Biomarkers	Calcium, Cholesterol in HDL, Creatinine, Magnesium, Potassium, Triglyceride, Total Cholesterol, Hemoglobin A1c, Glomerular Filtration Rate	Total Cholesterol, Cholesterol in HDL, Glomerular Filtration Rate, Urine albumin-creatinine ratio (UACR)*, Hemoglobin A1c*

eAppendix C: Analysis on race and ethnicity as predictors for our Weibull AFT model

The following eTable 5 presents the C-Index and Concordance Fractions for the Weibull AFT models that either include or exclude race/ethnicity as a predictor. Across nearly all scenarios, incorporating race and ethnicity improves the overall model performance and subgroup-specific performance. Due to these results, we include race and ethnicity as a predictor in our primary analyses.

eTable 5. Overall C-Index and subgroup-specific Concordance Fraction (CF) when excluding race and ethnicity as a predictor for our Weibull AFT model trained on all patients when tested on both all patients and patients without CVD.

Testing set	Model	C-Index	Sex CF		Race and Ethnicity CF		
			Female	Male	Non-Hispanic Black	Hispanic	Non-Hispanic White
All patients	Weibull	0.810	0.813	0.799	0.777	0.838	0.816
Patients w/o CVD history	Weibull	0.639	0.637	0.636	0.563	0.695	0.666
	Weibull w/o CVD history	0.657	0.653	0.656	0.619	0.775	0.643

eAppendix D: Comparing accuracy and fairness of our Weibull AFT model trained on patients without CVD history with PREVENT

Here, we analyze a version of our Weibull AFT model trained solely on patients without CVD history, which we refer to as Weibull AFT(-), and compare its performance with PREVENT. Overall, Weibull AFT(-) achieved a C-index of 0.646 when tested against patients without a history of CVD. The following eTable 6 shows that – even when trained on patients without CVD history – our model still outperforms PREVENT significantly in terms of fairness. Additionally, this model is better calibrated than PREVENT as shown in eFigure 3. Lastly, eFigure 4 shows that the Kidney disease-related features remain among the most important features.

eTable 6. Comparison of subgroup fairness metrics between our Weibull AFT model trained on patients without CVD history (Weibull AFT(-)) and PREVENT, when testing on patients without CVD history.

Metric	Model	Sex		Race and Ethnicity		
		Female	Male	Non-Hispanic Black	Hispanic	Non-Hispanic White
Concordance Fraction	Weibull AFT(-)	0.663	0.650	0.623	0.701	0.654
	PREVENT	0.568	0.569	0.541	0.600	0.572
Concordance Imparity	Weibull AFT(-)	0.005			0.067	
	PREVENT	0.002			0.058	

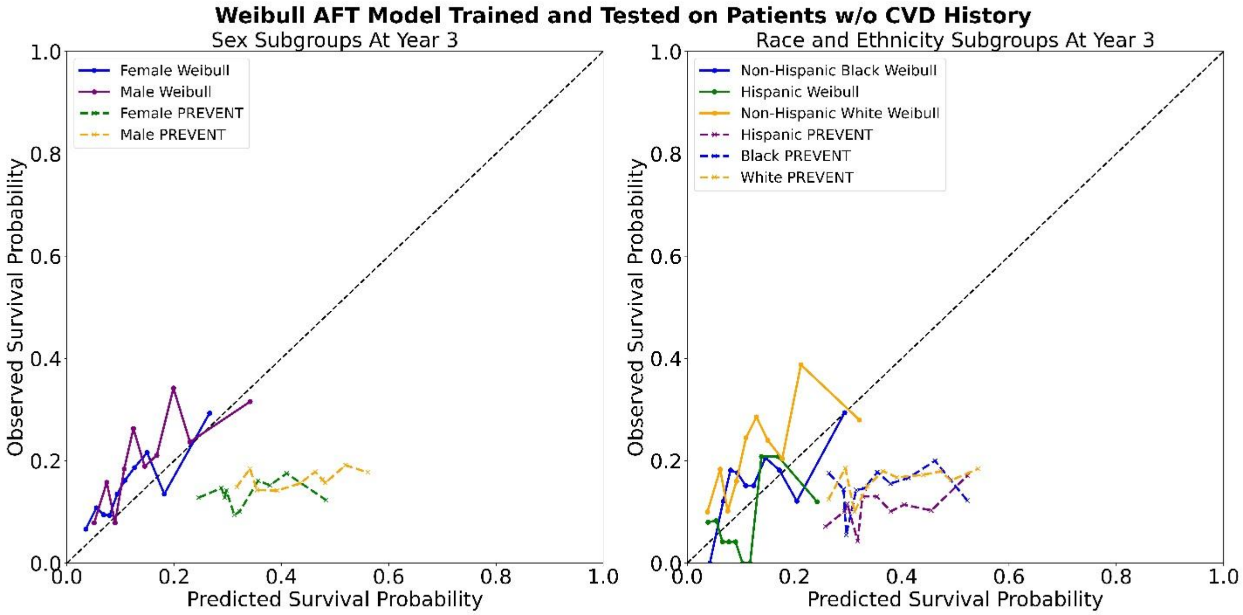

eFigure 3. Calibration plots of different sex subgroups and race and ethnicity subgroups comparing our Weibull AFT model trained on patients without CVD history and PREVENT, when testing on patients without CVD history. The ideal calibration is marked with black dashed line.

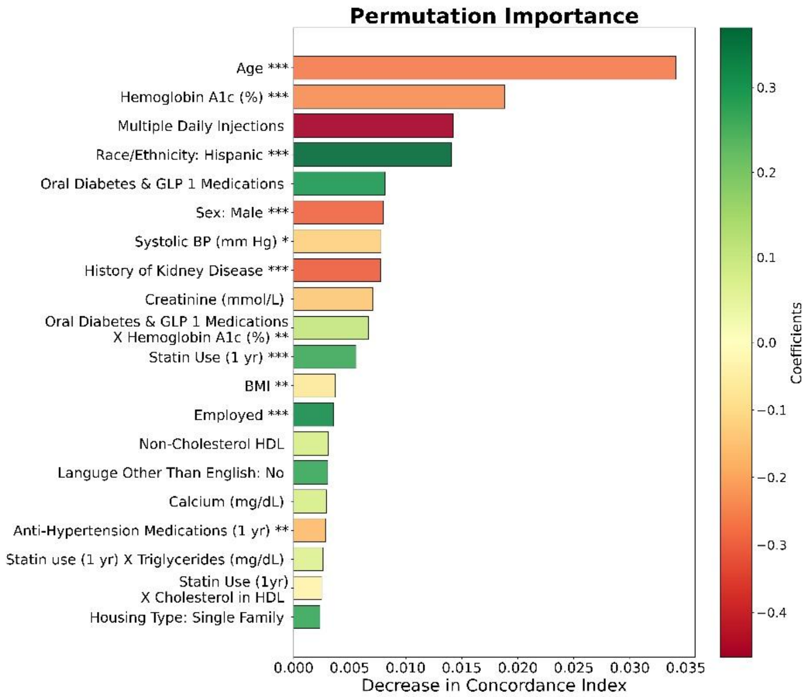

eFigure 4. Permutation importance plots of our Weibull AFT model trained on patients without CVD history and tested on patients without CVD history. We color-coded the predictors based on their variable coefficients to indicate their negative (red) or positive (green) effect on CVD risk. We mark the P-value of each predictor using *, where *** means P-value < 0.001, ** means P-value < 0.01, and * means P-value < 0.05.

eAppendix E: Converting PREVENT's 10-year risk to 3-year risk

Since PREVENT equations generate 10-year risk, we approximate its 3-year risk according to the equation:

$$r(3) = 1 - (1 - r(10))^{\frac{3}{10}},$$

where $r(t)$ represents the t-year risk generated by PREVENT. Note that $r(t) = 1 - S(t)$, where $S(t)$ is the survival function generated by PREVENT.

Derivation of formula and assumptions: The underlying assumption in our approximation is that for years t near 1-10, the PREVENT equations represent event times that are well-approximated by an exponential distribution with some baseline event rate λ. Accordingly, the cumulative hazard function is approximated by $H(t) = \lambda t$. This implies that $H(1) = \lambda$ so $H(t) = H(1)t$. Under this assumption, the survival function is given by $S(t) = \exp(-\lambda t) = \exp(-\lambda)^t = S(1)$, which can be rearranged to obtain our approximation equation above by substituting $r(3) = (1 - S(3))$ and $r(10) = \big(1 - S(10)\big)$.

Note that since the PREVENT equations are given by a Cox Proportional Hazards (CoxPH) model, this assumption is equivalent to taking the baseline hazard function in the CoxPH model to be $\lambda(t) = \lambda_0 t$ for some constant λ_0. Thus, we can set the event rate for the exponential model to be $\lambda = \lambda_0 \exp\,(-\beta^\top x)$ where β is the coefficient vector from the parametric portion of the CoxPH model and x is a vector of patient variables.
